# Effect of bone marrow-derived mesenchymal stem cells and stem cell supernatant on equine corneal wound healing in vitro

**DOI:** 10.1186/s13287-017-0577-3

**Published:** 2017-05-25

**Authors:** Amanda B. Sherman, Brian C. Gilger, Alix K. Berglund, Lauren V. Schnabel

**Affiliations:** Department of Clinical Sciences, North Carolina State University College of Veterinary Medicine, Raleigh, NC 27606 USA

**Keywords:** Mesenchymal stem cells, Equine, Corneal wound healing, Corneal stromal cells/ fibroblasts, Scratch assay, TGF-β1

## Abstract

**Background:**

We aimed to determine and compare the in vitro effects of autologous bone marrow-derived mesenchymal stem cells (BM-MSCs) and mesenchymal stem cell supernatant (MSC-Sp) on the wound healing capacity of equine corneal fibroblasts using a scratch assay.

**Methods:**

Bone marrow aspirates and eyes were collected from normal, euthanized horses with subsequent isolation and culture of BM-MSCs and corneal stromal cells. Corneal stromal cells were culture-expanded in the culture well of transwell plates and then treated with an autologous BM-MSC suspension (dose: 2.5 × 10^5^/100 μL media with the BM-MSCs contained within the insert well), MSC-Sp solution, or naive culture media (control) for 72 h. A linear defect in confluent cell cultures was created (i.e., corneal scratch assay) to assess the cellular closure (“healing”) over time. Three representative areas of the scratch in each culture were photographed at each time point and the scratch area was quantitated using image analysis software (ImageJ). Media from the scratches were analyzed for various growth factors using human enzyme-linked immunosorbent assay (ELISA) kits that crossreact with the horse.

**Results:**

There was a significant percentage decrease in the scratch area remaining in the BM-MSC and MSC-Sp groups compared to the control group. There was also a significant percentage decrease in the scratch area remaining in the BM-MSC group compared to the MSC-Sp group at 36 h post-scratch and all time points thereafter. The concentration of transforming growth factor (TGF)-β1 in the media was significantly higher in the BM-MSC group compared to the control group.

**Conclusions:**

The significant decrease in scratch area in equine corneal fibroblast cultures treated with autologous BM-MSCs compared to MSC-Sp or control treatments suggests that BM-MSCs may substantially improve corneal wound healing in horses. MSC-Sp may also improve corneal wound healing given the significant decrease in scratch area compared to control treatments, and would be an immediately available and cost-effective treatment option.

**Electronic supplementary material:**

The online version of this article (doi:10.1186/s13287-017-0577-3) contains supplementary material, which is available to authorized users.

## Background

Approximately 35% of all horses referred for ophthalmology specialty care are diagnosed with ulcerative keratitis [1]. Relative to other domestic species, horses are more susceptible to corneal injury, likely because of the increased surface area of their laterally placed, prominent globes, and their surrounding environment [2–4]. Positive bacterial and fungal cultures are common for horses diagnosed with ulcerative keratitis and have been previously reported in 84% and 40% of cases, respectively [5]. Injury may begin in the epithelial layer of the cornea; however, infectious organisms can activate corneal proteases that delay wound healing and then degrade corneal stroma which comprises the bulk of the corneal mass. This degradation of corneal stroma may ultimately lead to corneal perforation if not controlled [6]. Unfortunately, ulcerative keratitis is the most common equine ocular disease to result in enucleation due to this cascade of events [7]. Therefore, novel therapeutic interventions that accelerate corneal stromal wound healing and increase tensile strength and stability of such wounds are needed to help prevent blinding complications in ulcerative corneal disease. In addition, these therapies may also improve corneal clarity of the stroma and thus help to prevent re-injury.

Stem cells isolated from various sources have shown potential as a therapeutic agent in ocular regenerative medicine. Intravenously and locally infused blood-derived stem cells have been used clinically in horses to decrease inflammatory ocular disease and restore corneal strength [8]. Mesenchymal stem cells (MSCs) are a self-renewing population of multipotent cells that maintain the ability to differentiate into a variety of connective tissue cell types [9]. Ocular benefits of MSC therapy have also been documented [10–14]. MSCs used in rat and rabbit models of corneal injury have been shown to undergo direct, epithelial-like differentiation, increase endogenous cell survival and proliferation, accelerate corneal wound healing, and reduce oxidative stress [10–14]. MSCs also have the ability to target corneal damage and promote regeneration when injected intravenously [15]. Importantly, MSCs suppress proinflammatory cytokine profiles, decreasing opacification and neovascularization of the injured corneal surface [11]. Specifically, bone marrow-derived MSCs (BM-MSCs) accelerate corneal wound healing through faster re-epithelialization, decrease oxidative stress and inflammation, and increase corneal clarity more successfully than adipose-derived MSCs [10], and therefore are the focus of this study.

BM-MSCs are commonly used in equine practice to treat a variety of musculoskeletal injuries [16]. While long-term clinical trials still need to be performed for many of these injuries, there is both preclinical and clinical evidence to support the use of BM-MSCs for the treatment of tendon injuries [17–21], and there is growing evidence for the treatment of other soft tissue injuries including meniscal injuries [22]. In particular, studies of naturally occurring tendon injuries have shown a marked reduction in re-injury rate for horses treated with BM-MSCs compared to other conventional therapies [17, 21].

Although BM-MSCs are able to differentiate into a variety of mesenchymal tissue types in vitro, most in vivo studies in both the horse and other species have been unable to demonstrate long-term persistence or engraftment of these cells [23, 24]. These findings suggest that MSCs are improving healing mainly through paracrine mechanisms, and beg the question if the cells themselves are critical for therapy or if the potent cytokines that they secrete are enough to have a positive effect on healing [25–27]. Additionally, as corneal injuries require prompt treatment, an off-the-shelf acellular product without the concerns of immunogenicity would be an attractive therapeutic option rather than having to wait several weeks to culture autologous BM-MSCs or run the risk of a potential adverse event or decreased efficacy with allogeneic BM-MSCs [28, 29]. For these reasons, studying the influence of BM-MSC supernatant on wound healing is important when determining clinical therapeutic options for our patients.

MSCs may play an important therapeutic role in equine corneal wound healing given their numerous beneficial effects in both rat and rabbit corneal injury models [10–14, 25]. The purpose of this study is to determine the effect of autologous bone marrow-derived mesenchymal stem cell (BM-MSC) therapy and stem cell supernatant (MSC-Sp) on equine corneal wound healing capacity in vitro.

## Methods

**Fig. 1:**
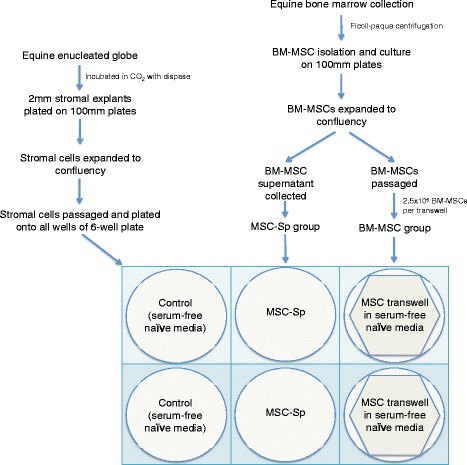
Flow chart of the study design for harvesting and culturing of autologous bone marrow-derived mesenchymal stem cells (*BM-MSCs*), bone marrow-derived mesenchymal stem cell supernatant (*MSC-Sp*) and equine corneal (stromal) cells

Please see Fig. 1 for an outline of the study design. All care and use of research animals in this study was approved and monitored by the North Carolina State University Institutional Animal Care and Use Committee (IACUC; protocol #14-180-O).

### Bone marrow collection and MSC isolation

Bone marrow aspirates were collected aseptically from the sternum of five horses (5 to 10 years of age) using an 11-gauge Jamshidi bone marrow biopsy needle following sedation and use of local anesthesia (2% lidocaine solution) or at the time of euthanasia. A total of 120 mL of marrow was collected into 60 mL syringes containing heparin. Purification of the bone marrow was performed using Ficoll-Paque Plus (GE Healthcare Bio-Sciences AB, Uppsala, Sweden) gradient centrifugation as previously described [30]. Cells were then plated on 100-mm tissue culture plates in low glucose (1 g/dL) Dulbecco’s modified Eagle’s medium (DMEM; Mediatech Inc., Manassas, VA, USA) containing 10% fetal bovine serum (FBS; Atlanta Biologicals, Inc., Flowery Branch, GA, USA), 2 mM l-glutamine (Mediatech Inc.), penicillin (100 units/mL; Mediatech Inc.), streptomycin (100 μg/mL; Mediatech Inc.), and basic fibroblastic growth factor (bFGF; 1 ng/mL; Mediatech Inc.). Cells were grown to confluency and passaged. To passage, 0.5% trypsin solution (Mediatech Inc.) was added to each well or plate and incubated at 37 °C in 5% CO_2_ for 5 min until cells detached. Media were added to each well or plate to stop the reaction and the cells and media was centrifuged for 5 min at 500 g in sterile 50-mL centrifuge tubes. The supernatant was removed and the cell pellet was suspended in media to plate onto 100-mm tissue culture plates for continued growth.

BM-MSCs were phenotyped for expression levels of a panel of surface markers using flow cytometry (CD29, CD44, MHC I, MHC II, LFA-1, CD90, and CD45RB). Dilutions of 1:200 (CD90), 1:100 (CD29 and CD44), 1:10 (MHC I, MHC II, and CD45RB), and neat (LFA-1) were used according to the manufacturer’s directions for commercial antibodies (CD29: EMD Millipore; CD44: BioRad; CD90: Washington State University; MHC I, MHC II, and LFA-1: Antczak Lab, Cornell University; CD45RB: Washington State University). Negative controls were only stained with secondary antibody and lacked a primary unconjugated antibody.

### Equine corneal cell isolation

Equine eyes were immediately enucleated (from the same five horses from which the bone marrow was collected) after euthanasia by intravenous administration of an overdose of sodium pentobarbital. Prior to euthanasia, horses underwent an ophthalmic examination including slit-lamp biomicroscopy and indirect ophthalmoscopy to ensure eyes were free of disease. After collection, globes were placed in 1% povidone iodine solution for 20 min, and then rinsed and immersed in cold phosphate-buffered saline (PBS) solution (pH, 7.2). Extraocular tissue was removed and globes were immersed in a Dispase II solution (5 mg Dispase/10 mL complete fibroblast media; Life Technologies Corporation, Grand Island, NY, USA) and incubated for 4 h at 37 °C in 5% CO_2_. After incubation, globes were again rinsed in PBS and the corneal epithelium was removed with a #15 Bard Parker scalpel blade. Two- to 3-mm corneal stromal explants were collected using a #15 scalpel blade and a 0.3-mm Colibri forcep. Explants were plated onto collagen-coated six-well plates or 100-mm plates and suspended in complete media: DMEM-F12 (Mediatech Inc.) supplemented with 5% FBS (Life Technologies Corp., Carlsbad, CA, USA), 200 IU/mL penicillin (Mediatech Inc.), 200 μg/mL streptomycin (Mediatech Inc.), 0.5 μg/mL amphotericin B (Mediatech Inc.), 20 ng/mL epidermal growth factor (EGF; Life Technologies Corp.), 5.6 μg/mL insulin (Sigma-Aldrich, Milwaukee, WI, USA), and 4 mM l-glutamine (Mediatech Inc.).

### Culture of equine corneal (stromal) cells

Culture media was changed every 2–3 days and cells were washed with sterile PBS before adding fresh media. Stromal explants were removed after 5–7 days once cells began to migrate. Stromal cells were allowed to grow to confluency (approximately 7–10 days) and then passaged in the same manner as the BM-MSCs and plated onto collagen-coated six-well plates. Corneal stromal cells were phenotyped for expression levels of a panel of positive (CD44, CD29, and CD90) markers using flow cytometry. Dilutions of 1:200 (CD90) and 1:100 (CD29 and CD44) were used according to the manufacturer’s directions for commercial antibodies (CD29: EMD Millipore; CD44: BioRad; CD90: Washington State University). Negative controls were only stained with secondary antibody and lacked a primary unconjugated antibody.

### In vitro wound healing experiment (scratch assay)

First-passage cultures of BM-MSCs and stromal cells were allowed to achieve 90% confluency, which took approximately 10–14 days. Half of the BM-MSC plates were again passaged as previously described, counted using an automatic cell counter, and plated onto transwell cell culture inserts (Costar, Corning Inc., Kennebunk, ME, USA) at a concentration of 2.5 × 10^5^ cells per well. Transwell inserts, the remaining confluent BM-MSC plates, and the collagen-coated six-well plate containing stromal cells were serum-starved overnight. Specifically, transwell inserts and BM-MSC plates were washed with PBS and incubated in BM-MSC culture media as described above, but without the addition of 10% FBS. Similarly, collagen-coated six-well plates with stromal cells were washed in PBS and incubated in stromal cell culture media as described above but without the addition of 5% FBS.

A scratch extending the length of each well was made on the cellular surface of each well of the six-well plate of stromal cells using a standard 200 μL pipette tip. Cells within the wound area were washed with PBS solution. Stromal cells of two wells of the six-well plate were then treated with 3 mL of culture media without serum (control group). Two other wells were treated with 3 mL of serum-free supernatant obtained from the BM-MSC plates (MSC-Sp treatment group). The two remaining wells were treated with 3 mL of culture media without serum combined with placement of the transwell insert containing 2.5 × 10^5^ of BM-MSCs (BM-MSC treatment group).

All plates were incubated at 37 °C in 5% CO_2_ for 72 h and media was not changed during this time. Three photomicrographs of each scratch were obtained at the initial time of wound creation and the same location was photographed every 12 h thereafter until completion of the study. Image analysis software (ImageJ, National Institutes of Health, Bethesda, MD, USA) was used to quantify (in pixels) the area of the wound remaining. This number was then converted to a percentage of the scratch area remaining at each time point.

### Measurement of EGF, PDGF-BB, and TGF-β1 concentrations

After completion of each scratch assay, 1.5 mL of supernatant was combined with 100 μL of protease inhibitor and stored at −80 °C until read for analysis. Concentrations of proteins related to ocular surface wound healing including EGF, platelet-derived growth factor (PDGF)-BB, and transforming growth factor (TGF)-β1 were measured for each group of each horse. Growth factors were measured using previously validated human enzyme-linked immunosorbent assay (ELISA) kits (EGF and PDGF-BB: R&D Systems, Minneapolis, MN, USA; TGF-β1: Promega, Madison, WI, USA) that crossreact with the horse.

### Data and statistical analysis

Parametric normally distributed data (i.e., area of scratch, cytokine concentrations) were compared for each group using one-way analysis of variance (ANOVA) models with individual comparison student's *t* test analysis. Differences were considered significant at *p* < 0.05. All results and probabilities were calculated using computerized statistical software (JMP 10, SAS Inc. Cary, NC, USA).

## Results

### Study population

Five horses were included in the study. The mean age of the horses was 6.6 ± 2 years (range 5 years to 10 years). Three horses were mares and two were geldings. Breeds of horses included Thoroughbred (2/5, 40%), Quarterhorse (1/5, 20%), Shetland Pony (1/5, 20%), and Peruvian (1/5, 20%). No horse had evidence of ocular or periocular disease prior to euthanasia.

### Stromal cell and BM-MSC analysis with flow cytometry

**Fig. 2:**
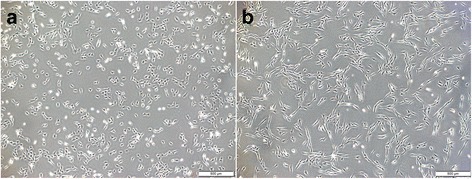
Frozen corneal stromal cells and BM-MSCs were thawed and plated in respective media at 10,000 cells/cm^2^. Twenty-four hours after plating, stromal cells (**a**) and BM-MSCs (**b**) were imaged via phase microscopy. Magnification 4×, *scale bar* = 500 μm

**Fig. 3:**
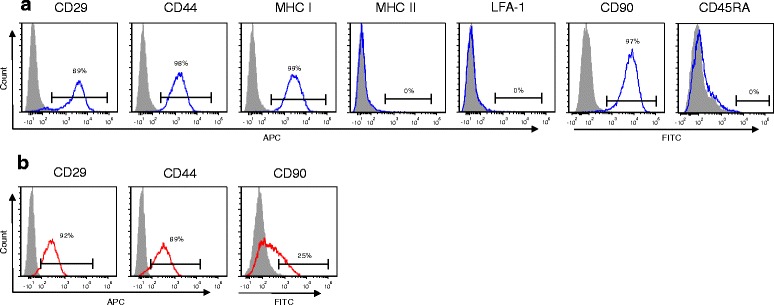
Representative histograms of cell surface marker expression on BM-MSCs (**a**) and corneal stromal cells (**b**) from one horse. All horses had similar flow cytometry results

The morphological comparison of stromal cells and BM-MSCs is shown in Fig. 2. Corneal stromal cells from stromal explants and BM-MSCs were phenotyped with flow cytometry. All BM-MSCs were positive for CD29, CD44, CD90, and MHC I, and negative for LFA-1 and CD45RB. All corneal stromal cells were positive for CD29 and CD44, while CD90 expression was heterogeneous (Fig. 3). Expression of CD29, CD44, and CD90 was higher on BM-MSCs than on corneal stromal cells.

### Scratch assays

**Fig. 4:**
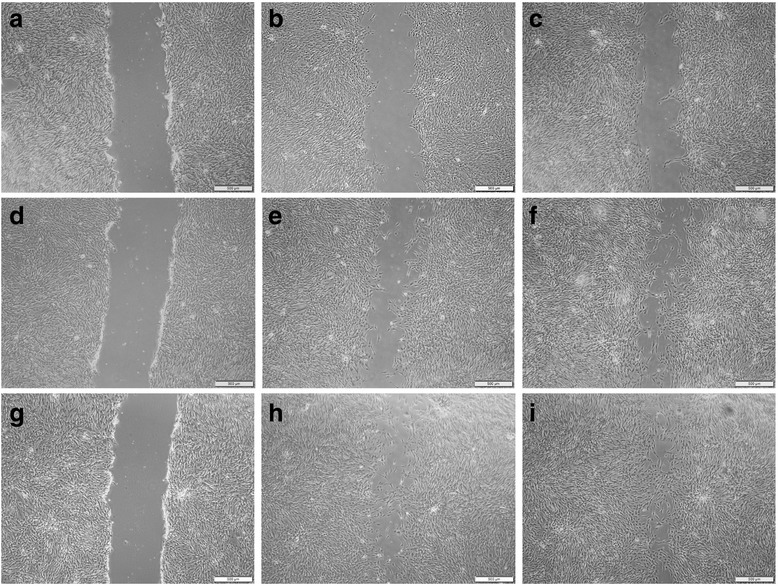
Photomicrograph of initial linear defect (scratch) created with a 200 μl pipette tip compared with same scratch at time 36 h post scratch and at end of study (72 h) for each experimental group. Magnification 4×, *scale bar* = 500 μm. **a**–**c** Photomicrographs of the same horse from the control group at time 0, 36 h, and 72 h, respectively. **d**–**f** Photomicrographs of the same horse from the MSC-Sp group at time 0, 36 h, and 72 h, respectively. **g**–**i** Photomicrographs of the same horse from the BM-MSC group at time 0, 36 h, and 72 h, respectively

**Fig. 5:**
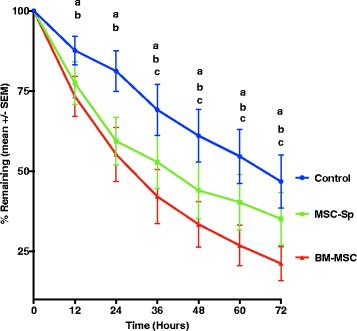
Graphical comparison of the mean ingrowth (depicted as the percentage of original scratch width remaining) and standard error of the mean (*SEM*) of corneal stromal cells from the scratch assay among the control, mesenchymal stem cell supernatant (*MSC-Sp*), and bone marrow-derived mesenchymal stem cell (*BM-MSC*) groups. ^a^ *p* ≤ 0.014, between control and MSC-Sp groups; ^b^ *p* ≤ 0.0002, between control and BM = MSC groups; ^c^ *p* ≤ 0.034, between MSC-Sp and BM-MSC groups; *n* = 5 with two replicates per horse

Scratch assays were performed on the stromal cells obtained for each of the five horses in the study with two replicates per horse (Fig. 4). No significant difference was found regarding initial scratch width at time 0 among the three groups (control, BM-MSC, and MSC-Sp) for any horse in the study. Results of the scratch assay are summarized in Fig. 5. An additional graph showing individual data points for all of the horses is provided as Additional file 1. At time points 12, 24, 36, 48, 60, and 72 h post-scratch, the MSC-Sp group had a significant decrease in scratch area remaining compared to the control group (*p* = 0.0076, 0.0004, 0.0023, 0.0012, 0.0050, and 0.0140, respectively). Additionally, at time points 12, 24, 36, 48, 60, and 72 h post-scratch, the BM-MSC group had a significant decrease in scratch area remaining compared to the control group (*p* = 0.0002 at 12 h and *p* < 0.0001 for all remaining time points). The BM-MSC group did not have any significant difference in wound healing compared to the MSC-Sp group until 36 h post-scratch. At this time point, and continuing at time points 48, 60, and 72 h post-scratch, the BM-MSC group had significantly less scratch area remaining compared to the MSC-Sp group (*p* = 0.0346, 0.0346, 0.0063, and 0.0026, respectively). On average, at 72 h post-scratch, the control group had 46.8 ± 18.4% of the scratched area remaining, the MSC-Sp group had 35.2 ± 18.3% of the scratched area remaining, and the BM-MSC group had 21.3 ± 11.9% of the scratched area remaining. No scratch was 100% healed by the end of the 72-h experiment.

### ELISA supernatant analysis

**Fig. 6:**
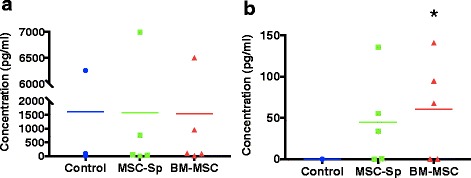
Graphical representation of culture supernatant EGF (**a**) and TGF-β1 (**b**) concentrations determined by ELISA for the control, mesenchymal stem cell supernatant (*MSC-Sp*), and bone marrow-derived mesenchymal stem cell (*BM-MSC*) groups. Individual data points for all horses; bar represents the mean for each group. The concentration of TGF-β1 in the BM-MSC group was significantly higher (*p* = 0.0057) than the control group as indicated by the asterisk; *n* = 5 with two replicates per horse

The concentration of the growth factors PDGF, EGF, and TGF-β1 in the media of each group was evaluated. PDGF was not detected in the media of any group in our study. EGF and TGF-β1 results are shown in Fig. 6. EGF was positively identified in the control (1604 ± 2778 pg/mL), MSC-Sp (1568 ± 2801 pg/mL), and BM-MSC (1531 ± 2573 pg/mL) groups for all horses; however, no significant difference in EGF concentration was noted among the three groups (*p* = 0.9968). TGF-β1 was not identified in the control group for any horse. TGF-β1 was positively detected in both the BM-MSC (61 ± 71 pg/mL) and MSC-Sp (45 ± 59 pg/mL) groups. The concentration of TGF-β1 in the BM-MSC group was significantly higher than the concentration of TGF-β1 in the control group (*p* = 0.0057). While TGF-β1 was also identified in the MSC-Sp group, the concentration of this growth factor in this media was not significantly higher (*p* = 0.0504) than the concentration of TGF-β1 in the control group nor was the concentration significantly different from the BM-MSC group (*p* = 0.6489).

## Discussion

Bone marrow-derived mesenchymal stem cell (BM-MSC) therapy is a novel, therapeutic option for the acceleration of corneal healing in the horse. In our in vitro study, at 72 h after scratch initiation, we documented a significant percentage decrease in the scratch area remaining between the BM-MSC and MSC-Sp groups compared to the control group. Additionally, the BM-MSC group showed improved healing compared to the MSC-Sp group as there was a significant percentage decrease in the scratch area remaining between the BM-MSC and MSC-Sp groups at 36 h post-scratch and all time points thereafter. The cytokine TGF-β1 was found in significantly higher concentrations in the BM-MSC group compared to the control group, but not when compared to the MCS-Sp group.

It is expected that both BM-MSC and MSC-Sp would have a positive influence on wound healing, as demonstrated in this study. It is known that MSCs secrete soluble factors to exert influence over surrounding tissue [31] and the beneficial effects of BM-MSCs, therefore, are believed to be due in large part to the potent paracrine factors that the cells secrete, which are both anti-inflammatory and immunomodulatory [26, 27, 32]. These interactions likely also play a role in paracrine signaling to host cells, for example in the cornea, to promote repair of local tissue. It has been previously shown that MSC-conditioned media has a therapeutically positive effect on tissue repair without the use of whole cells [33]. This likely explains why a beneficial effect in the MSC-Sp group was demonstrated. Although this effect was not as significant as when there was direct biological feedback between cell types, this result strengthens the argument for use of a stem cell-derived but cell-free product clinically, which may prove beneficial due to its lack of immunogenicity, ease of storage, and preferred routes for administration.

Despite their potent immunomodulatory effects, BM-MSCs can still be immunogenic due to their constitutive surface expression of major histocompatibility complex (MHC) class I and variable surface expression of MHC class II [34]. MHC class II proteins are potent alloantigens leading to recognition by alloreactive CD4^+^ T cells and promotion of T-cell proliferation [35], and it has been shown that MHC class II proteins are upregulated by inflammatory mediators including interferon (IFN)-γ [34]. Recent studies in the horse have shown that allogeneic donor BM-MSCs that are MHC-mismatched to the recipient cause both cellular and humoral immune responses in the recipient [28, 34] whether they are MHC class II negative or positive, suggesting that recipient recognition of foreign MHC class I alone is capable of causing a significant immune response. Furthermore, antibodies present in the recipient antisera following donor BM-MSC injection are capable of killing donor BM-MSCs in vitro through the complement cascade suggesting that allogeneic BM-MSCs may have a limited therapeutic effect [29].

It is important to note that the normal cornea is considered an immune privileged site due to lack of corneal vasculature and lymphatics, low numbers of local antigen-presenting cells, high concentrations of immunomodulatory cytokines, and low constitutive expression of MHC class I on corneal cells [36]. Therefore, it is unclear what effect, if any, allogeneic BM-MSCs would have on immune responses in this predominantly immune-tolerant environment. Damage to the surface epithelium can result in impairment of normal immune tolerance, however, leading to recruitment of antigen-presenting cells to the limbus, production of inflammatory cytokines, antigenic stimulation, and upregulation of MHC class II [37]. For these reasons, it is reasonable to still recommend the use of an autologous as opposed to an allogeneic source in a wounded corneal environment, as used in this study. This, unfortunately, is not ideal for acute corneal wounds as autologous BM-MSCs can take weeks to culture and achieve numbers for use. Ideally, a supernatant product or the use of an allogeneic source that is MHC class I low and MHC class II negative would be preferred. Further studies are needed to evaluate the safety and efficacy of various allogeneic BM-MSCs donors.

Determining the specific soluble factors that BM-﻿MSCs secrete can help us to further clarify and define their role in tissue repair. Therefore, we analyzed the media from each treatment group at the end of the study for peptide growth factors typically found and influential in healing of the injured equine corneal environment, including EGF, PDGF-BB, and TGF-β1. Previous studies have evaluated the efficacy of certain exogenous growth factors towards the acceleration of wound healing. A study by Haber et al*.* documented the positive proliferative effects of exogenous EGF and PDGF-BB on both equine corneal epithelial cells and keratocytes in vitro [38]. EGF has been shown to increase cell proliferation and chemotactic migration [39, 40], and PDGF-BB increases matrix production and chemotaxis and enhances inflammatory reactions to accelerate tissue repair [38, 41]. EGF was a component of the naive fibroblast media and serum-free media and, therefore, its detection in all groups in this study was expected. EGF was, however, not found in a higher concentration in our treatment groups compared to the control population, indicating it was not upregulated by BM-MSCs during wound healing within the context of this assay. PDGF-BB was not found in any group in our study.

TGF-β1 was not expressed in the control group; however, increased concentrations of TGF-β1 were noted in both the BM-MSC and MSC-Sp groups. This is supported by previous studies which demonstrated higher TGF-β1 expression of MSCs compared to limbal stem cells [42] and higher expression of TGF-β1 in rat corneas treated with MSC therapy [43]. TGF-β1 has been shown to induce connective tissue growth factor from fibroblasts [44, 45] which is important for fibroblast proliferation and extracellular matrix component production, including collagen. TGF-β1 has also been shown to stimulate integrin expression which is involved in acceleration of wound repair [46]. However, in the study by Haber et al. exogenous TGF-β1 had a negative effect on proliferation of corneal epithelial cells and keratocytes [38]. Therefore, its effects on wound healing are variable and warrant further investigation. Additionally, the increased concentration of TGF-β1 in our study was only significant for the BM-MSC group compared to the control group. We suspect the MSC-Sp group would have gained significance with an increased population of horses studied.

An additional goal of our study was to determine the phenotype of the corneal cells cultured, as we strived to create an in vitro environment as similar to the wounded equine cornea as possible, and to compare and differentiate them from a BM-MSC phenotype. Through flow cytometry, our corneal cells were determined to be of fibroblastic morphology. Fibroblasts are an appropriate model for this project as keratocytes undergo fibroblastic transformation after injury and are therefore the primary cell type in wound repair [47]. Our primary culture media was supplemented with 5% FBS, which likely explains the fibroblastic phenotype. It has been previously shown that equine primary cultures generated from stromal explants plated in media supplemented with 10% FBS selectively induce fibroblasts instead of keratocytes [48] which is in agreement with our study.

Limitations of this study include the small number of horses. It is possible additional horses may strengthen the conclusions of this study. Additionally, there was variability in the number of passages fibroblasts underwent prior to the scratch assay as this was dependent on how quickly each horse’s BM-MSCs took to reach confluence. Variability among horses was also noted in the proliferative capabilities of fibroblasts which may have influenced our results. Lastly, we do not yet know what concentration of BM-MSCs is optimal for having the best effect on stromal cells.

Future goals of this research would include in vivo studies to prove efficacy of topical products for clinically affected epithelial and stromal lesions. Prior to these studies, it would be important to optimize our stem cell product by determining the concentration of cells that maintain the most positive therapeutic influence. Additionally, it has been previously shown that MSCs of passage 4 have an increased wound healing potential compared to cells only undergoing two passages [49]. The BM-MSCs in our study underwent two passages prior to placement on the transwell inserts and, therefore, it would be beneficial to know if continued passages of these cells would result in faster wound healing.

## Conclusions

In conclusion, in this population of equine eyes, we were able to show that autologous bone marrow-derived MSCs and MSC-Sp are capable of accelerating fibroblast healing of a scratch assay in vitro and that BM-MSCs lead to faster healing compared to MSC-Sp. In vivo studies are warranted to allow us to continue to evaluate BM-MSC safety and efficacy for corneal wound healing.

## Additional file

Additional file 1: Graphical depiction of scratch assay data for control, MSC-Sp, and BM-MSC groups for all horses displayed as individual data points. (PDF 31 kb)

## Abbreviations

BM-MSC: Bone marrow-derived mesenchymal stem cell
DMEM: Dulbecco’s modified Eagle’s medium
EGF: Epidermal growth factor
ELISA: Enzyme-linked immunosorbent assay
FBS: Fetal bovine serum
MHC: Major histocompatibility complex
MSC: Mesenchymal stem cell
MSC-Sp: Mesenchymal stem cell supernatant
PBS: Phosphate-buffered saline
PDGF: Platelet-derived growth factor
TGF: Transforming growth factor
