# Supplementary figures and images for: Effect of bone marrow-derived mesenchymal stem cells and stem cell supernatant on equine corneal wound healing in vitro
Source: Stem Cell Res Ther. 2017 May 25;8:120. doi: 10.1186/s13287-017-0577-3 (PMC5445363; doi:10.1186/s13287-017-0577-3)

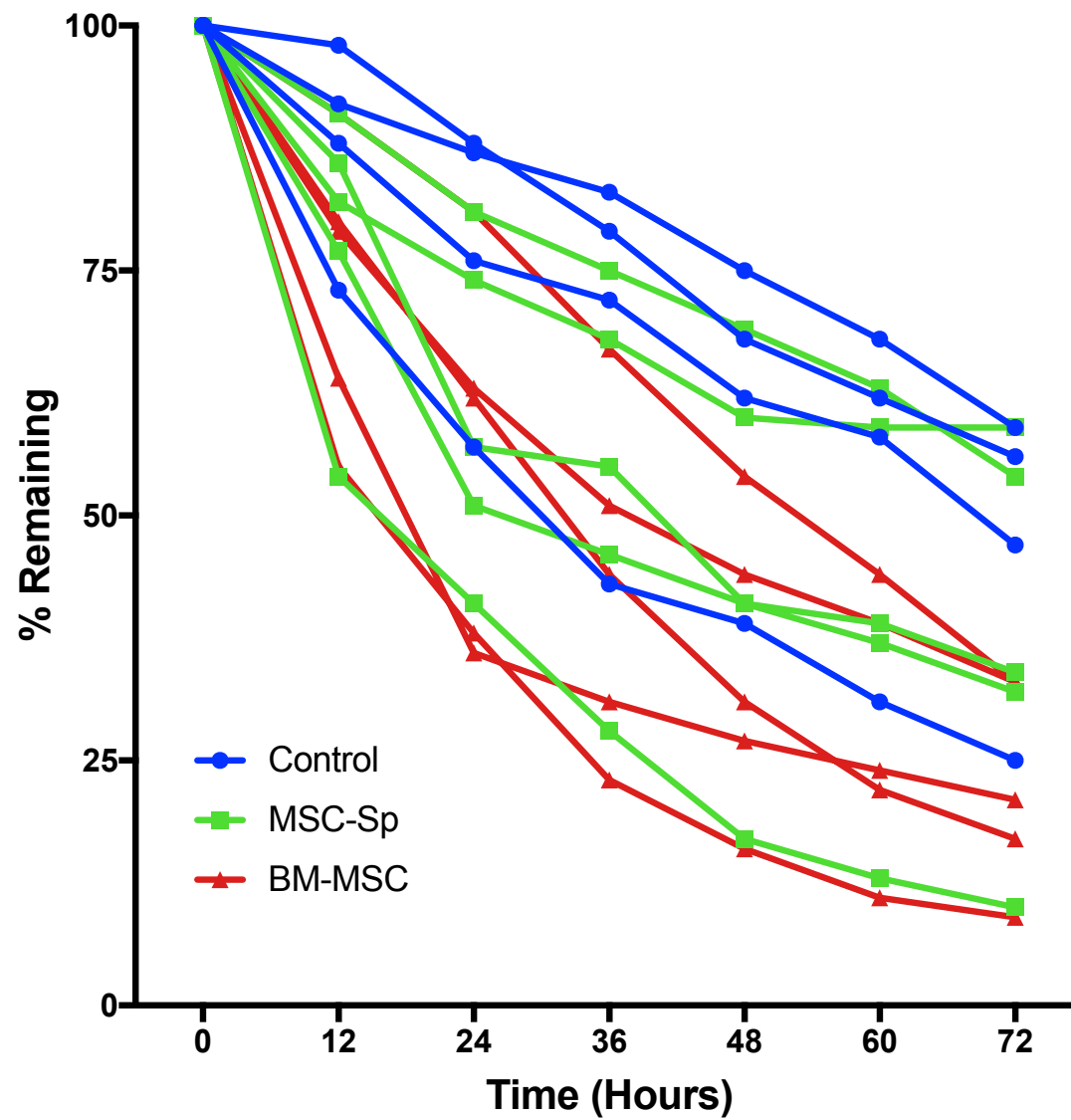

Supplement: Additional file 1: — Graphical depiction of scratch assay data for control, MSC-Sp, and BM-MSC groups for all horses displayed as individual data points. (PDF 31 kb) [file 13287_2017_577_MOESM1_ESM.pdf]
